# Rare variant of *HSPG2* is not involved in the development of adolescent idiopathic scoliosis: evidence from a large-scale replication study

**DOI:** 10.1186/s12891-019-2402-x

**Published:** 2019-01-15

**Authors:** Chao Xia, Leilei Xu, Bingchuan Xue, Fei Sheng, Yong Qiu, Zezhang Zhu

**Affiliations:** Department of Spine Surgery, The Affiliated Drum Tower Hospital of Nanjing University Medical School, Nanjing, China

**Keywords:** *HSPG2*, Rare variation, Adolescent idiopathic scoliosis, Replication

## Abstract

**Background:**

Rare variants of *HSPG2* have recently been reported to function as a potential contributor to the susceptibility of adolescent idiopathic scoliosis (AIS) in the Caucasians. A replication study in the different population is warranted to validate the role of *HSPG2* in AIS. The aim of this study was to determine the association between *HSPG2* and AIS in the Chinese patients and to further investigate its influence on the phenotype of the patients.

**Methods:**

SNVs p.Asn786Ser of *HSPG2* was genotyped in 1752 patients and 1584 normal controls using multiple ligase detection reactions. The mRNA expression of *HSPG2* in the paraspinal muscles was quantified for 90 patients and 26 controls. The The Student’s t test was used to analyze the inter-group comparison of the *HSPG2* expression. The relationship between the *HSPG2* expression and the curve magnitude of the patients was analyzed by the Pearson correlation analysis.

**Results:**

No case of mutation in the reported SNV p.Asn786Ser of *HSPG2* was found in our cohort. The mRNA expression of *HSPG2* in patients was comparable with that in the controls (0.0016 ± 0.0013 vs. 0.0019 ± 0.0012, *p* = 0.29). 42 patients with curve magnitude > 60 degrees were assigned to the severe curve group. The other 58 patients were assigned to the moderate curve group. These two groups were found to have comparable *HSPG2* expression (0.0015 ± 0.0011 vs. 0.0017 ± 0.0014, *p* = 0.57). And there was no remarkable correlation between the expression level of *HSPG2* and the curve severity (*r* = 0.131, *p* = 0.71).

**Conclusions:**

*HSPG2* gene was not associated with the susceptibility or the phenotypes of AIS in the Chinese population. The whole *HSPG2* gene can be sequenced in more AIS patients to identify potentially causative mutations.

## Background

Adolescent idiopathic scoliosis (AIS) is a complex spinal deformity that affects millions of children worldwide [1]. To benefit the prognosis and therapeutic strategy for patients with AIS, it is crucial to have a clear understanding of the etiopathogenesis of this disease. For the past half century, numerous studies have been carried out to investigate the etiology of AIS while the conclusions remain obscure [2]. Traditionally, AIS is considered as a multifactorial disorder that involves interaction between different factors, including genetic susceptibility, growth disturbance, hormones and metabolic dysfunction, abnormal central nervous system and proprioception impairment [3–7]. Among these factors, previous familial studies of AIS have strongly implied the genetic background of AIS [8, 9]. But to date, the exact inheritance mode of AIS remains unclear.

Confined by the low efficiency to define the genetic markers, whole-genome linkage analysis was firstly used in the genetic research of AIS which could only identify large chromosome regions potentially containing disease-related genes [10, 11]. Gradually, it became feasible to analyze SNPs through TaqMan real-time quantitative PCR assay, which was a high-throughput method to differentiate the genotype of target variants. Since then, multiple susceptible genes of AIS have been discovered through candidate genetic association studies [12–17]. To be noted, however, few of these genes could be successfully replicated in different populations, which greatly weakened the reliability of such candidate susceptible genes in AIS [18, 19]. Commonly, ethic differences and small sample size of the patients were considered to underlie the discrepancy between the original and the replication studies [18, 19]. In addition, the speculation based on which those candidate genes was selected was mostly lack of essential scientific evidence, thus inevitably leading to the failed replication.

To overcome the above-mentioned limitations of candidate genetic association studies, a much powerful tool, genome-wide genotyping chip, was utilized to identify the susceptible gene of AIS [20–26]. Sharma et al. [26] performed the first genome-wide association study (GWAS) in the Caucasians and reported that CHL1 was associated with AIS. In the following decade, more susceptible loci of AIS were discovered in the Caucasian, the Japanese and the Chinese populations through GWAS, including LBX1, GPR126, BNC2, PAX1, LBX1-AS1, BCL2, AJAP1, PAX3, TNIK, MEIS1, and MAGI1 [20–25]. However, only a small proportion of the heritability of AIS can be explained by these common variants.

With the advances of sequencing techniques, more research teams have begun to analyze the role of rare variants in AIS using whole exome sequencing (WES) in recent years [27–29]. With a low minor allele frequency in normal population, rare variants are usually expected to have larger impact on the inheritance of complex disease than common variants. Patten et al. [27] performed the first WES in AIS and reported 3 rare variants of the POC5 functionally associated with AIS. Subsequently, Buchan et al. [28] reported that rare variants in FBN1 and FBN2 could contribute to both risk and severity of AIS. Haller et al. [30] found that rare variants across extracellular matrix genes contributed strongly to the risk of AIS in patients with European ancestry. Overall, identification and validation of rare variants associated with AIS is a promising method to explain the missing heritability of this complex disease.

In a recent study, rare variants of *HSPG2* were reported to function as a potential contributor to the susceptibility of AIS in the Caucasians [31]. Obviously a replication study in the different population is warranted to validate the role of *HSPG2* in AIS. This study aimed to determine the association between *HSPG2* and AIS in the Chinese patients and to further investigate its influence on the phenotype of the patients.

## Methods

### Subjects

Under the approval of the local institutional review board, a cohort of 1752 female AIS patients and 1584 controls were included in the current study. All the patients were excluded to have neurological defect through MRI examination. All the controls were excluded to have scoliosis through Adam’s Forward Bend Test using the standard criteria performed by a senior spine surgeon (Z.Z.). Demographic data were collected for each participant, including initial age, curve pattern and curve magnitude measured by the Cobb angle method on the standing posteroanterior X-ray films.

### Genotyping of the rare variant

All the subjects signed the informed consent for the collection of blood samples. Genomic DNA was then extracted with the commercial kit (Qiagen K.K., Tokyo, Japan). Single nucleotide variant (SNV) p.Asn786Ser (rs143736974) of *HSPG2* was genotyped for all participants. Allelic-specific multiple ligase detection reactions (LDR) was used for genotyping assay as previously reported, with the primer shown in Table 1. Fifteen percent of the samples were randomly selected for Sanger sequencing to ensure the reproducibility of the LDR results.

**Table 1:** Primers for the genotyping assay and expression analysis

	Primers
p.Asn786Ser	
TC	TTTTTTTTTTTTTTTTGTTGCACTGTGGCCCCTCCtTGC
TT	TTTTTTTTTTTTTTTTTTTGTTGCACTGTGGCCCCTCCtTGT
TR	TGTGCTGGCAATTCTAGAAGAAGGAtttttt
GAPDH	TTTTTTTTTAGCTGCAGATCCTGCACCAAGAGC
Forward	5′- GAGTCAACGGATTTGGTCGT − 3’
Reverse	5′- TTGATTTTGGAGGGATCTCG − 3’
HSPG2	
Forward	5′ - TACACACGCCACCTGATCTC -3’
Reverse	5′ - GCTGCCAGTAGAAGGACTCA −3’

### Genotyping of common variations in HSPG2

Common variants covering *HSPG2* gene were identified through the Ensemble database (http://www.ensembl.org/index.html). GWAS database that was composed of 1446 AIS patients and 2080 controls was processed with the PLINK (version 1.90, http://zzz.bwh.harvard.edu/plink/tutorial.shtml) to determine the genotyping results of these common variants.

### RNA extraction and real-time qPCR

Ninety-eight female AIS patients were included in the expression analysis of *HSPG2*. Twenty-eight female lumbar disc herniation (LDH) patients with no presence of spinal deformity were included as the controls. The paraspinal muscle was collected from each subject during the surgery. For AIS patients, we collected the muscle sample from both the concave side and convex side at the apex of the curve. Total RNA was then extracted from the muscle tissue using a commercial kit (CWBio. Co. Ltd). The mRNA expression of *HSPG2* was quantified with real-time polymerase chain reaction (PCR) on ABI 7900HT, with GAPDH used as the endogenous control. All amplifications were performed in triplicate. The relative expression of *HSPG2* was normalized using the ΔΔCt method. The primers of *HSPG2* and GAPDH were shown in Table 1.

### Statistical analysis

SPSS version 17.0 (SPSS Inc., Chicago, USA) was used for data analysis. Cochran-Armitage trend test was used to calculate the association between common variants and AIS. Patients included expression analysis were classified into different subgroups according to the curve pattern or the curve severity. The Student’s t test was used to analyze the inter-group comparison of the baseline characteristics and mRNA expression of *HSPG2*. The relationship between the *HSPG2* expression and the curve magnitude of the patients was analyzed by the Pearson correlation analysis. The statistical was set at *p* < 0.05.

## Results

### Demographic data

The mean age of patients included in the LDR analysis was 15.3 ± 3.4 years (range 10.5–18.8 years). The mean curve magnitude was 54.3 ± 11.2 degrees (range 27–72 degrees). One thousand eighteen patients had curve magnitude more than 50 degrees and thus underwent posterior spinal correction surgery. The mean age of the healthy controls was 18.9 ± 3.2 years (range 16.5–22.8 years).

### Genotyping of SNVs

No case of mutation in the reported SNV p.Asn786Ser of *HSPG2* was found in our cohort. All the 3336 subjects were found to have genotype TT. A total of 16 SNPs covering *HSPG2* were analyzed, with the distribution of minor allele frequency summarized in Table 2. All the SNPs were found to have comparable allele frequency between the patients and the controls.

**Table 2:** The allele frequency of 16 SNPs covering HSPG2

SNP	MA	MAF		p	OR (95% CI)
		Patients (*n* = 1446)	Controls (*n* = 2080)		
rs4654770	T	0.170	0.169	0.9262	1.01 (0.90–1.12)
rs12040859	T	0.190	0.192	0.8902	0.99 (0.87–1.11)
rs17459139	T	0.165	0.167	0.8384	0.99 (0.87–1.11)
rs7556412	G	0.179	0.195	0.1463	0.90 (0.81–0.99)
rs12043008	G	0.179	0.175	0.7164	1.03 (0.92–1.14)
rs10799718	A	0.385	0.371	0.278	1.06 (0.94–1.17)
rs11810496	G	0.393	0.390	0.8497	1.01 (0.90–1.12)
rs2445142	G	0.395	0.381	0.3187	1.06 (0.94–1.17)
rs878949	T	0.148	0.151	0.8051	0.99 (0.87–1.11)
rs2501257	A	0.395	0.386	0.5053	1.04 (0.94–1.16)
rs6680566	C	0.394	0.388	0.6465	1.03 (0.92–1.14)
rs6698486	G	0.347	0.341	0.6487	1.03 (0.92–1.14)
rs9426783	C	0.443	0.436	0.62	1.03 (0.92–1.14)
rs4654773	T	0.440	0.433	0.5781	1.03 (0.92–1.14)
rs16826053	C	0.199	0.189	0.383	1.06 (0.94–1.17)
rs10917067	T	0.107	0.116	0.2869	0.91 (0.82–1.01)

*MA* indicates minor allele, *MAF* indicates minor allele frequency, *OR* indicates odds ratio, *CI* indicates confidential interval

### mRNA expression level of HSPG2

A total of 98 patients and 28 controls were included for expression analysis. Eight patients and 2 controls were excluded from the analysis due to degradation of total RNA. The expression level of *HSPG2* was successfully detected in the paraspinal muscles of 90 patients and 26 controls. There was no significant difference regarding the mean age of the two groups (15.2 ± 1.1 years vs. 15.5 ± 1.2 years, *p* = 0.48). The mRNA expression of *HSPG2* in patients was comparable with that in the controls (0.0016 ± 0.0013 vs. 0.0019 ± 0.0012, *p* = 0.29).

### Relationship between HSPG2 and phenotype of the patients

For the 90 patients included in the expression analysis, 42 patients had curve magnitude more than 60 degrees, thus assigned to the severe curve group. Forty-eight patients with curve magnitude less than 60 degrees were assigned to the moderate curve group. These two groups classified by curve severity were found to have comparable *HSPG2* expression (0.0015 ± 0.0011 vs. 0.0017 ± 0.0014, *p* = 0.57). And there was no remarkable correlation between the expression level of *HSPG2* and the curve severity (*r* = 0.131, *p* = 0.71), either.

As for the curve pattern, 67 patients had single thoracic curve and the other 23 patients had double major curve. There was no significant difference of *HSPG2* expression between the curve pattern groups (0.0017 ± 0.0012 vs. 0.0014 ± 0.0013, *p* = 0.31).

## Discussion

Genetic findings of AIS have been greatly hindered by its clinical and genetic heterogeneity. Previous association studies have revealed many disease-related genes, most of which however appeared inconclusive as indicated by the following replication studies [18, 32, 33]. Carried out in different populations, replication studies have failed to validate the reported associations of ER-α, ER-β, MTNR1b, MATN1, TPH1, IGF1, MMP3, IL6 and TGF-β with AIS [18, 19, 32, 34]. To the best of our knowledge, LBX1 and PAX1 are the only two susceptible genes that could be successfully validated in Caucasians and Asians with a minimum sample size of over 3000 patients [20, 35]. The statistical power regarding the associations between these genes and AIS has reached the genome-wide significance that was defined as a *p* value of less than 5.0 × 10^− 8^. Obviously, a large sample size and a rigorous setting of statistical power are key factors to ensure reliable and reproducible findings in genetic studies.

Another difficulty of AIS genetic research lies in that the reported common variants can only explain a small amount of AIS heritability. All the susceptible variants reported by previous GWASs of AIS had an OR value between 1 and 2, leaving over 90% of the AIS heritability unexplained [20–26]. Since low frequency variants with large effect sizes could be involved in the missing heritability, the role of rare variants in the development of AIS has recently become a widely investigated topic [27–32]. Baschal et al. [31] performed WES in a four-generation idiopathic scoliosis (IS) family and identified the SNV p.Asn786Ser of *HSPG2* gene as a novel causative variant of IS. Enrichment of this mutation was then validated in two independent cohorts composed of 241 IS patients [31]. To validate the association of *HSPG2* with the development of AIS, we performed the genotyping of p.Asn786Ser in a large cohort consisted of 1752 patients and 1584 controls. No case of mutation was found in this cohort as all the subjects presented the same genotype TT. As for common variants covering *HSPG2*, none of the 16 SNPs had remarkably different allele frequency between the patients and the controls. Moreover, we observed comparable *HSPG2* expression in the paraspinal muscles between the patients and the controls. To summarize, our findings did not support the association of *HSPG2* with the susceptibility of AIS in Chinese population.

*HSPG2* gene, which has 94 exons, encodes perlecan which binds to basement membrane proteins such as collagen and laminin and to cell surface receptors [36]. Homozygous *HSPG2* mutations could lead to a more severe phenotype in human disease, such as Schwartz- Jampel syndrome Type 1 and Dyssegmental Dysplasia, Silverman-Handmaker Type [37, 38]. Schwartz-Jampel syndrome is reported to be associated with kyphoscoliosis [39]. Baschal et al. [31] therefore speculated that haploinsufficiency of *HSPG2* may be associated with a progressive IS and acted as a potential contributor to the phenotype. Aiming to clarify the relationship between *HSPG2* and phenotypes of the patients, we classified patients into different subgroups according to the curve pattern or the curve severity. We found that *HSPG2* expression was not associated with either the curve severity or the curve pattern. Herein, it appeared that *HSPG2* had no effect on the phenotype of the AIS patients.

It is unlikely for the current study to have false-negative results as our sample size is large enough to detect the potential mutation. The susceptibility genes of AIS may play important roles in the diagnosis and therapy of AIS in the future. We believe that the role of *HSPG2* in the development of AIS still needs further investigation. The primary limitation of our study lies in that we did not sequence all the exons of *HSPG2*. It cannot be excluded that enrichment of other mutations of *HSPG2* could play a role in AIS. Besides, the function of p.Asn786Ser should be investigated through in-vivo cellular experiment to clarify its role in AIS.

## Conclusions

*HSPG2* gene was not associated with the susceptibility or the phenotypes of AIS in the Chinese population. In future study, functional studies of p.Asn786Ser is warranted to clarify whether this variant can regulate the expression of *HSPG2*. The whole *HSPG2* gene can be sequenced in more AIS patients to identify potentially causative mutations.

## Abbreviations

AIS: Adolescent idiopathic scoliosis
GWAS: Genome-wide association study
*HSPG2*: Human heparan sulfate proteoglycan 2
LDR: Ligase detection reactions
SNV: Single nucleotide variant
WES: Whole exome sequencing
